# Incidence of obstetric anal sphincter injuries following breech compared to cephalic vaginal births

**DOI:** 10.1186/s12884-023-05595-5

**Published:** 2023-05-04

**Authors:** Perrine Leborne, Renaud de Tayrac, Zakarya Zemmache, Chris Serrand, Pascale Fabbro-Peray, Lucie Allegre, Emmanuelle Vintejoux

**Affiliations:** 1 Department of Obstetric Gynecology, University Hospital Arnaud de Villeneuve, 371 avenue du Doyen Gaston Giraud - 34000, Montpellier, 34000 France; 2 Department of Obstetric Gynecology, University Hospital of Nimes, Place du Pr R. Debré, NIMES CEDEX9, 30029 France; 3 Statistics department (BESPIM), University Hospital of Nimes, Place du Pr R. Debré, NIMES CEDEX9, 30029 France

**Keywords:** Breech delivery, Obstetrical anal sphincter injuries, OASIs, OASI, Pelvic floor, Perineal injuries, Perineum

## Abstract

**Introduction:**

Obstetric anal sphincter injuries (OASIs) at the time of childbirth can lead to serious consequences including anal incontinence, dyspareunia, pain and rectovaginal fistula. These types of lesions and their incidence have been well studied after cephalic presentation deliveries, but no publications have specifically addressed this issue in the context of vaginal breech delivery. The goal of our study was to evaluate the incidence of OASIs following breech deliveries and compare it with cephalic presentation births.

**Methods:**

This was a retrospective cohort study involving 670 women. Of these, 224 and 446 had a vaginal birth of a fetus in the breech (breech group) and cephalic (cephalic group) presentations respectively. Both groups were matched for birthweight (± 200 g), date of delivery (± 2 years) and vaginal parity. Main outcome of interest was to evaluate the incidence of OASIs following breech vaginal birth compared to cephalic vaginal births. Secondary endpoints were the incidence of intact perineum or first-degree tear, second-degree perineal tear and rates of episiotomies in each group.

**Results:**

There was no statistically significant difference in OASIs incidence between the breech and cephalic groups (0.9% vs. 1.1%; RR 0.802 (0.157; 4.101); p = 0.31). There were more episiotomies in the breech group (12.5% vs. 5.4%, p = 0.0012) and the rate of intact or first-degree perineum was similar in both groups (74.1% vs. 75.3%, p = 0.7291). A sub-analysis excluding patients with episiotomy and history of OASIs did not show any statistically significant difference either.

**Conclusion:**

We did not demonstrate a significant difference in the incidence of obstetric anal sphincter injuries between women who had a breech vaginal birth compared to cephalic.

## Key message

Breech vaginal birth does not seem to be associated with a higher risk for obstetric anal sphincter injuries compared to a cephalic vaginal birth.

## Introduction

Obstetrical Anal Sphincter Injuries (OASIs) are complex perineal tears that can occur at the time of vaginal birth and may have negative consequences on a woman’s short-term and long-term functional outcomes. These consequences include anal incontinence, dyspareunia, pain and rectovaginal fistula [1–3]. Moreover, a significant number of OASIs are not diagnosed immediately after delivery and hence not repaired or managed properly, increasing the risk of such complications [4]. Therefore, recognition and repair of this type of trauma has been the focus of several quality improvement and training programs in recent years. Tears involving the ano-rectal complex have been extensively researched in relation to cephalic vaginal births. However, they have not been specifically studied in the context of vaginal breech deliveries. Moreover, interventions to reduce the risk of OASIs have been, relatively, well studied at the time of head and shoulder expulsion in case of a fetus in the cephalic presentation. Based on these studies, some obstetrical techniques are recommended for perineal protection, such as a manual control of the expulsion of the head and support of the posterior perineum [5, 6]. However, there are no recommendations about prevention of perineal injuries during a vaginal breech delivery, except that the episiotomy is not routinely recommended [5, 7, 8].

During a breech delivery, expectant management is recommended at the time of breech expulsion [7] to mitigate the risk of a nuchal position of the arm. However, during delivery of the shoulders and head, there are no recommendations regarding whether to perform active maneuvers or continue with expectant management [9, 10]. Nonetheless, if there is a delay, maneuvers are recommended to avoid prolonged compression of the umbilical cord and cervical retraction [11, 12]. Contrary to cephalic presentation, the use of maneuvers to expedite head expulsion in a breech delivery does not allow time for the physiological moulding to occur. Second, there is the risk of additional distension of the introitus by the accoucheur’s hands whilst performing these maneuvers. Third, the perineal stretch that happens at the time of the delivery of the breech and shoulders is minimal compared to that occurring at crowning in a cephalic birth [13], which can have an impact on the decision to perform an episiotomy, its accuracy and results in the quick delivery of the largest diameters of the fetus through a sub-optimally stretched perineum. Therefore, it is plausible to believe that a vaginal breech delivery is associated with a higher risk of OASIs compared to a cephalic vaginal birth.

To date, no studies have evaluated the risk of OASIs, as a primary outcome, in the context of breech vaginal births. Therefore, the main aim of this study was to evaluate the rate of OASIs following breech compared to cephalic vaginal deliveries.

## Materials and methods

This study was approved by Institutional Review Board (IRB) n°18.10.02 (INDS n°0414121119 - Ethics committee of South Mediterranean) and all patients gave informed consent.

### Design and population

We conducted a retrospective cohort study at two university hospitals in France. The sample comprised of women of any parity who had a spontaneous vaginal delivery between 2012 and 2020 and 2014–2020 in the first and second centers respectively. Multiple gestations were excluded from the study.

All the women who had a vaginal breech delivery in the studied periods were included and categorized as “breech group”. For every included breech delivery two cephalic births who fulfilled our inclusion criteria and matched for date of delivery (± 2 years), birthweight (± 200 g) and vaginal parity were randomly selected after matching. Owing to the lack of data, we used probability proportional to size sampling without replacement method for the matching. These pregnancies constituted the “cephalic group”.

Using the participating units’ electronic patient record systems, data were gathered on maternal age at birth, vaginal parity, BMI, previous history of perineal trauma, gestational diabetes in the index pregnancy, gestational age at delivery, the type of labor (spontaneous or induced), total duration of labor, duration of the second stage of labor, birthweight, history of previous perineal trauma and the degree of any perineal trauma sustained at the birth under review.

### Care pathway

Women diagnosed with a breech presentation at term were managed in line with the French national guidelines where they were encouraged to have a vaginal birth if the estimated fetal weight was < 3800 g and there was no evidence of fetal neck hyperextension on ultrasound scan performed at the beginning of spontaneous labor. Radiopelvimetry was not mandatory to assess suitability for a breech vaginal birth [14]. Epidural analgesia was highly recommended and continuous fetal monitoring was routinely performed. The total duration of labor was calculated from the onset of labor (defined as regular uterine contractions associated with cervical dilatation) till delivery of the baby. While the duration of the second stage of labor was defined as the time between the diagnosis of full cervical dilatation and delivery of the baby.

For a breech delivery a senior obstetrician, senior anesthetist, trainee obstetrician and the neonatology team were routinely present at birth. Either the senior or trainee obstetrician conducted the delivery. The use and type of intrapartum maneuvers undertaken were left to the accoucheur’s discretion and these were mostly Lovset’s and Bracht’s, Mauriceau’s or Vermelin’s maneuvers [15]. Routine episiotomy was not recommended for perineal protection.

Cephalic presentation deliveries were mostly performed by midwives. Manual perineal protection and a selective episiotomy policy were followed in both participating units following the national recommendations [16, 17].

Postnatally, birth attendants routinely and systematically assessed the perineum and any identified trauma was classified and managed as per international recommendations [5, 18, 19]. In case of any uncertainly about the assessment, a second opinion was sought from the senior obstetrician.

### Outcome measures

Our main outcome of interest was to evaluate the incidence of OASIs following breech vaginal birth compared to cephalic vaginal births. Additionally, we were interested to explore the incidence of intact perineum or first-degree perineal tear, second-degree tear and episiotomy in each group.

### Statistical analysis

Based on the published literature, we considered the baseline rate of OASIs in cephalic vaginal birth to be 1.25% [20]. Due to the lack of reported OASIs rate in breech vaginal delivery in the literature, we hypothesized that the relative risk of OASIs following breech vaginal birth compared to cephalic to be 4. To demonstrate this level of difference, at a power of 80% and a two-sided alpha risk of 5%, 693 patients would be needed. We decided to include 2 cephalic births for each breech vaginal delivery, hence, 231 breech and 462 cephalic vaginal births were required.

The statistical analysis was conducted by BESPIM, Nimes University Hospital using SAS (SAS Institute, Cary, NC, USA) version 9. Summary statistics were used to describe demographic and clinical variables. In between group differences were assessed using the Chi-square test or Fisher’s exact test for categorical variables and t-test or Mann-Whitney test for continuous variables. All tests were 2-tailed and statistical significance was defined as a p value of < 0.05.

## Results

During the studied periods there were a total of 228 singleton breech vaginal births. Of these, 2 had instrumental deliveries and 2 had a previous breech birth during the study period and could not be included twice, leaving 224 patients included in the breech cohort. Based on the breech to cephalic inclusion ratio of 1 to 2 and our *a priori* set matching criteria, we should have had 448 women identified from our database and included in the cephalic cohort. However, two women in the breech delivery group could only be matched with one cephalic delivery each. Therefore, the number of women included in the cephalic cohort and in the study in total were 446 and 670 women respectively.

Our population demographics are presented in Table 1. Among patients who delivered in cephalic presentation, 371 (97.12%) delivered in an occiput anterior position and 11 (2.88%) in a posterior occiput position. There were no statistically significant differences between both groups with regards to body mass index (BMI), birthweight, date of birth, term deliveries, vaginal parity or gestational diabetes in the index pregnancy. The only significant difference was in the mean maternal age at delivery between the breech and cephalic groups (30.51 [± 5.28] vs. 29.05 [± 5.84] years, p = 0.0017).

**Table 1:** Population demographics

	Breech N = 224	Cephalic N = 446	*P*
**BMI**			
Median IQR (q1; q3) Range (min;max) Missing data	24.81 21.68; 27.83 15.62; 53.35 20	24.14 21.45; 27.89 15.42; 47.67 17	0.4192
**Maternal Age**			
Mean (SD)	30.51 (5.28)	29.05 (5.84)	0.0017
**Gestational age**			
Median IQR (q1; q3) Range (min;max) Missing data	39 38; 40 34; 42 6	39 38; 40 34; 41 20	0.679
**Birthweight in grams**			
Mean (SD)	3018.7 (436.73)	3022.91 (426.78)	0.9049
**Gestational diabetes**			
Yes	13 (5.8%)	38 (8.5%)	0.211
No	211 (94.2%)	408 (91.5%)	
**Vaginal Parity**			
1	107 (47.8%)	214 (48%)	0.9998
2	58 (25.9%)	115 (25.8%)	
3	36 (16.1%)	72 (16.1%)	
4	23 (10.3%)	45 (10.1%)	

Mat.: Maternal, GA: Gestational age, BW: Birthweight, GD: Gestational diabetes, SD: Standard deviation

Labor was significantly more likely to be induced in the cephalic cohort (P < 0.0001). Of note, till 2018 breech inductions of labor were not authorized by our national guidelines. The second stage was significantly longer in association with breech vaginal deliveries (p = 0.0145) (Table 2).

**Table 2:** Labor and perineal outcomes

	Breech N = 224	Cephalic N = 446		p
**Labor**				
Spontaneous	215 (96%)	346 (77.8%)	< 0.0001	
Induced	9 (4%)	99 (22.2%)		
Missing data	0	1		
**Total duration of labor (min)**				
Median	375,5	339	0.876	
IQR (q1; q3)	219; 495	230; 490		
Range (min; max)	31; 958	18; 1077		
Missing data	74	213		
**Duration of 2nd stage of labor**				
Median	83.00	49.00	0.0145	
Q1, Q3	22.00; 140.00	13.00; 117.00		
Range (min; max)	3.00; 287.00	1.00; 280.0		
Missing	81	267		
**History of perineal tears**				
No	138 (61.9%)	275 (61.7%)	0.955	
Yes	85 (38.1%)	171 (38.3%)		
Missing data	1	0		
**Perineal tears**				
OASIs	2 (0.9%)	5 (1.1%)	0.77	
Episiotomy	28 (12.5%)	24 (5.4%)	0.001	
1st degree or no tear	166 (74.1%)	336 (75.3%)	0.73	
2nd degree tear	28 (12.5%)	81 (18.2%)	0.06	

One patient (0.22%) in the cephalic group gave a previous history of a 3rd degree perineal tear but none in the breech cohort. In the reviewed birth, OASIs was reported in 2 (0.89%) and 5 (1.12%) women in the breech and cephalic groups respectively. There was no statistically significative difference between the incidence of OASIs in each group (RR 0.802 (0.157–4.101); p = 0.31) (Table 2). All the OASIs were 3rd degree tears, where three were classified as 3a, one as 3b and 3 were just labeled as 3rd degree tears (Table 3).

**Table 3:** Characteristics of participants sustaining an OASIs

Type of OASIs	BMI	Vaginal parity	Age at delivery	History of OASIs	Gestational age	Presentation	Labor	Total duration of labor in minutes	Duration of second stage of labour in minutes	Birthweight in grams
3A	21.8	1	32	No	38^+ 6^	Breech	Spontaneous	459	59	3000
3A	25.6	1	28	No	41	Breech	Spontaneous	386	86	3610
3B	23.8	1	30	No	40^+ 5^	Cephalic	Spontaneous	735	268	3250
3A	27	3	24	No	41^+ 4^	Cephalic	Induced	350	50	3550
3	19	1	25	No	39^+ 6^	Cephalic	Spontaneous	90	-	2670
3	19.6	1	25	No	40^+ 4^	Cephalic	Spontaneous	420	-	3670
3	25.8	1	23	No	40^+ 2^	Cephalic	Spontaneous	-	-	3660

Additionally, we conducted two post hoc subgroup analysis excluding patients who had an episiotomy and with previous history of OASIs due to the potential confounding effect of these variables. The subgroup analysis excluding patients undergoing an episiotomy did not show a significant difference (RR 0.869 (0.170–4.440); p = 0.32). Similarly, there was no significant difference in the subgroup analysis after exclusion of the patient with a previous history of OASI (RR 0.997 (0.185–5.375); p = 0.33).

Regarding the secondary outcomes, the rate of episiotomy was higher in the breech group (12.5%, n = 28) compared to the cephalic one (5.4%, n = 24) (p = 0.001). In contrast, there were more second-degree perineal tears in the cephalic compared to the breech group (18.16%, n = 81 vs. 12.5%, n = 28). However, this difference did not reach statistical significance (p = 0.06). The rate of intact perineum or 1st degree tear was comparable between both groups (74.1% [n = 166] for breech and 75.3% [n = 336] for cephalic, p = 0.72). These results are summarized in Table 2.

## Discussion

Although, there have been several observational studies exploring maternal and fetal outcomes following vaginal breech deliveries [15, 21], to our knowledge, this is the first study conducted with the primary aim of exploring the risk of OASIs following such deliveries. Our findings showed that the overall incidence of OASIs in the studied population was 1%, which is comparable to previous reports. In 2010, Blondel et al. reported an OASIs rate of 0.5% following spontaneous vaginal births in France [17]. However, Thubert et al. suggested that the rate increased after that due to better recognition of these injuries. Indeed, the latest perinatal survey reported an overall OASIs rate of 0.8% in 2016 [20].

The 0.9% OASIs rate identified in our breech delivery cohort is lower than what we anticipated. Roman et al. conducted a large retrospective cohort study involving 15,818 women who delivered of a fetus in breech presentation between 1987 and 1993 [17]. Amongst those who had breech vaginal delivery (n = 5897), they reported third- and fourth-degree tear rates of 1.0% and 0.1% respectively, representing an OASIs rate of 1.1%. Bogner et al. [19] compared severe perineal injuries following vaginal breech deliveries in “all-fours” position and classical support and reported rates of 58.5% with classical support compared to 14.6% in all fours position. Nevertheless, they included episiotomies in their definition of severe injuries. Importantly, they only reported 2 patients with grade 3 lacerations. While, in a cohort of 269 women, Louwen et al. [21] reported third and fourth degree tears rates of 4.9% and 1.7% following vaginal breech deliveries in the dorsal and upright positions respectively. More recently, Benmessaoud et al. reported an OASIs rate of 3.4% following spontaneous labor progression in breech presentation in 203 women [15]. However, none of these studies were designed to evaluate perineal injuries as their primary outcome.

With regards to other perineal outcomes, the rates of intact perineum or first degree tears identified in our cohort are comparable to those reported by Benmessaoud et al. (74.1% vs. 72.4%) [15]. Similarly, the rate of second-degree perineal tear in our study was 12.5%, which is almost similar to the 14.1% rate reported by Louwen et al. [21] Moreover, our episiotomy rate in the breech group of our study concurs with that reported by Benmessaoud et al. [15] in their nulliparous cohort (13.1%) and with Louwen et al. in their group of women delivering in the dorsal position (10.0%). However, our rate was much higher than the 0.9% rate reported by Louwen and associates in their “upright position” group [21]. In contrast, the episiotomy rate in the breech vaginal birth cohort is much lower than the 34.1% overall rate of episiotomy reported by Bogner et al. study [19].

This study did not show any statistically significant difference in the rate of OASIs between cephalic (1.1%) and breech vaginal deliveries (0.9%) nor the rates of other birth-related perineal injuries with the exception of episiotomies. Nonetheless, it is important to emphasize that despite the higher rate of episiotomy following breech (12.5%) compared to cephalic (5.4%) deliveries, it is much lower than the rates reported in other studies. Indeed, our rate was less than the 2009–2010 AUDIPOG episiotomy rate with breech deliveries of 28.4% [22]. Furthermore, according to Clesse et al., the rate of episiotomy in the overall population in France had stabilized at around 30% in 2013 [23]. This rate was in line with the 2005 national French guidelines, which recommended a reduction in episiotomy rates to 30% [5]. Interestingly, our two university hospitals seem to have a very conservative practice with regards to episiotomy, compared to national background figure.

The matching criteria used for our study groups were date of delivery, birthweight and vaginal parity. Other confounding variables not included in our matching criteria include operative vaginal delivery, advanced maternal age, history of OASIs, type of episiotomy, occipital posterior position and a prolonged second stage of labor [20].

Operative vaginal deliveries were one of the exclusion criteria in our study to abolish their effect as a confounder. Moreover, we conducted two post hoc sub-analyses excluding patients with episiotomy and with a previous history of OASIs to mitigate the risk of bias introduced by these variables.

Finally, the median maternal age in both of our study (30.51 [± 5.28] and 29.05 [± 5.84] years) and the small number of babies born in the occipital posterior position make it very unlikely that neither of these factors would have affected our results.

Although there was no statistically significant difference in the OASIs rates between both of our cohorts, the rates of some variables, known to be associated with a higher risk of OASIs, were significantly higher in our breech delivery group; namely episiotomy and duration of the second stage of labor. Episiotomy was reported to be a risk factor for OASIs in a meta-analysis by Pergialotis et al., [24] with an odds ratio of 3.69 (1.45–9.38, p < 0.001). Furthermore, the second stage of labor was significantly longer in the breech (83 min) compared to the cephalic group (49 min) (p = 0.0145) which might directly be linked to the management of breech delivery where obstetricians are more likely to allow longer second stage in breech presentation to mitigate the risk of head entrapment. Smith and colleagues [25] reported a higher rate of OASIs in association with a prolonged second stage (adjusted OR 1.49 (1.13–1.98)). Despite these factors the OASIs rate was not higher in the Breech cohort. It is possible that this discrepancy is a reflection of the accuracy with which episiotomies are cut in our units, that the prolonged second stage duration was still below 120 min or a result of a small sample size. However, exploring the reasons behind this observation is beyond the scope of our work and our proposed possibilities are merely speculative.

Although this study was not powered to assess potential risk factors for OASIs in the breech cohort, it is interesting that our data still demonstrated a trend for the presence of some of the risk factors known to be associated with significant degrees of perineal trauma. Indeed, the women who sustained an OASIs at the time of a breech vaginal birth had a higher mean birthweight and a longer duration of second stage. These factors are known to be significantly associated with OASIs [24, 26]. It is also worth noting that both patients who sustained an OASIs were nulliparous and neither of them had an episiotomy.

Undoubtedly, being the first study to explore the risk of OASIs following breech vaginal birth as its primary outcome adds strength to our work. However, we appreciate that the retrospective nature of our design and the number of data that are missing for some of our secondary outcomes are limitations to our work. Additionally, our sample size was based on an assumed relative risk of 4 for OASIs following breech compared to cephalic birth. Given the much lower event rate and its proximity to the rate in the comparator group, our study would be underpowered to demonstrate an existing smaller difference. Therefore, for future studies exploring a similar research question, it would be prudent to base the sample size on an assumed relative risk of OASIs with breech birth of 2, or even lower, compared to cephalic deliveries. It is also possible that relying on clinical examination for diagnosing OASIs, rather than imaging, could be perceived as a limitation. However, the use of ultrasound scan in the immediate postpartum period to assess sphincter integrity is neither routinely used nor recommended in clinical practice [27, 28, 3, 29]. Moreover, in both of the participating units, there is strong emphasis on postnatal systematic perineal assessment and all midwives and obstetricians receive regular structured training in line with international recommendations [4].

## Conclusion

Our study did not show any statistically significant differences in OASIs rates between breech and cephalic vaginal delivery. Although we recommend that our findings should be interpreted with caution, due to the above stated limitations, our results provide a degree of reassurance to clinicians and women about breech vaginal birth and risk of perineal trauma.

## Data Availability

the datasets used and/or analysed during the current study are available from the corresponding author on reasonable request.
